# Local Boards of Health in England

**Published:** 1858-01

**Authors:** 


### LOCAL BOARDS OF HEALTH IN ENGLAND.

THERE are now in England no less than one hundred and ninety-six local Boards of Health, all of which have been established since the cholera outbreak of 1848. Many of these have borrowed money for their works, which will soon be returned with interest.

